# Teste de Degrau de Seis Minutos como Alternativa para a Avaliação da Capacidade Funcional de Pacientes com Doenças Cardiovasculares

**DOI:** 10.36660/abc.20210252

**Published:** 2021-05-06

**Affiliations:** 1 Universidade Nove de Julho São PauloSP Brasil Programa de Pós-Graduação em Ciências da Reabilitação - Universidade Nove de Julho, São Paulo, SP - Brasil; 2 Universidade Federal Rural de Pernambuco Departamento de Educação Física RecifePE Brasil Departamento de Educação Física da Universidade Federal Rural de Pernambuco, Recife, PE - Brasil; 3 Universidade Federal de Pernambuco RecifePE Brasil Programa de Pós-Graduação em Educação Física da Universidade Federal de Pernambuco, Recife, PE - Brasil

**Keywords:** Hipertensão, Pressão Arterial, Hereditariedade/genética, Exercício, Esportes, Futebol, Endotélio, Atletas

A capacidade funcional é um importante marcador de morbimortalidade em pacientes com doenças cardiopulmonares.^1^^,^^2^ Embora a medida direta do consumo pico de oxigênio (VO_2pico_) por meio dos testes cardiopulmonares (padrão-ouro) seja o método mais indicado para avaliar a capacidade funcional, a sua utilização na prática clínica ainda é restrita, devido ao custo elevado.^1^^,^^2^

O teste de degrau de seis minutos (TD6M), além de ser rápido, tem como principal vantagem a necessidade de um espaço mínimo para a sua realização. Esses fatores, e o fato de não necessitar sinais sonoros, torna-se bastante atrativo para a utilização em clínicas e hospitais. Os testes com degrau são utilizados há muito tempo para avaliar a capacidade funcional, tanto em pessoas saudáveis como naqueles com doenças pulmonares.^3^ Nos pacientes com doenças pulmonares, observou-se uma correlação positiva (r = 0,76) entre o TD6M e o teste de caminhada de seis minutos,^4^ que necessita de maior espaço para sua realização.^5^ Curiosamente, os dados de correlação entre o TD6M e a medida direta da capacidade funcional são escassos, sobretudo em pacientes com doenças cardiovasculares.

O estudo Ritt et al.^6^ busca suprir essa lacuna ao submeter 171 pacientes com insuficiência cardíaca e doença arterial coronariana ao TD6M e ao teste cardiopulmonar em esteira. Os resultados indicaram correlação significante entre VO_2pico_, obtida no teste de esteira, e o desempenho no TD6M (de r = 0,69). Ademais, elaborou-se uma equação preditora de estimativa do VO_2pico_ para homens [VO_2pico_ = 19,6 + (0,075* TD6M) – (0,10*idade)] e para mulheres [VO_2pico_ = 19,6 + (0,075*6 TD6M) - (0,10*idade) – 2], baseadas nos resultados do teste do degrau. Por fim, os autores identificaram 105 subidas como ponto de corte para VO_2pico_ acima de 20 ml/kg*min, algo que em pacientes cardíacos é considerado um bom indicador de prognóstico cardiovascular.

Apesar dos resultados interessantes, devemos destacar aspectos que merecem ser elucidados em estudos futuros. Faltam indicadores psicométricos importantes do TD6M, como a reprodutibilidade e a sensibilidade à mudança. A identificação de pontos de corte é altamente relevante e aplicável na prática clínica. No entanto, para a identificação de indivíduos com baixa aptidão funcional, convém elaborar novos pontos, que considerem idade, sexo e estatura, entre outros fatores. Pontos de corte baseados em uma amostra única e heterogênea possivelmente identificam sobretudo os pacientes com idade mais avançada e do sexo feminino. No entanto, é possível que ao serem comparados aos pares da mesma idade e sexo, os mesmos apresentem capacidade funcional dentro do esperado. Tais aspectos vêm sendo amplamente discutidos no teste de caminhada de seis minutos e podem ser considerados para o TD6M.^7^^–^^9^

Em suma, o trabalho de Ritt et al.^6^ apresenta evidencias iniciais e interessantes da utilização do TD6M para avaliar a aptidão funcional de pacientes com doença arterial coronariana e insuficiência cardíaca. Por se tratar de um teste com grande potencial de utilização na prática clínica, estudos futuros sobre o TD6M como marcador prognóstico e suas características psicométricas, bem como valores de referência de acordo com o sexo e a idade, serão bem-vindos.
