# Supplementary material for: Teste de Degrau de Seis Minutos como Alternativa para a Avaliação da Capacidade Funcional de Pacientes com Doenças Cardiovasculares
Source: Arq Bras Cardiol. 2021 May 6;116(5):896–7. [Article in Portuguese] doi: 10.36660/abc.20210252 (PMC8121458; doi:10.36660/abc.20210252)
Supplement: Translation

# Translation: The Six-Minute Step Test as an Alternative for Functional Capacity Assessment in Patients with Cardiovascular Diseases

**Affiliations:** 1 São PauloSP Brazil Programa de Pós-Graduação em Ciências da Reabilitação - Universidade Nove de Julho, São Paulo, SP - Brazil; 2 RecifePE Brazil Departamento de Educação Física da Universidade Federal Rural de Pernambuco, Recife, PE - Brazil; 3 RecifePE Brazil Programa de Pós-Graduação em Educação Física da Universidade Federal de Pernambuco, Recife, PE - Brazil

**Keywords:** Hypertension, Blood Pressure, Heredity/genetic;, Exercise, Sports, Football, Endothelium, Athletes

Functional capacity is an important morbimortality marker in patients with cardiopulmonary diseases.^1^^,^^2^ Although the direct measurement of peak oxygen consumption (VO_2peak_), using cardiopulmonary tests (the gold standard), is the most adequate method for assessing functional capacity, its use in clinical practice is still restricted due to its high cost.^1^^,^^2^

The 6-minute step test (6MST), in addition to being fast, has as its main advantage the need for minimum space for its performance. These factors, added to the fact that it does not require audible signals, become very attractive for its use in clinics and hospitals. Step tests have been used for a long time to assess functional capacity, especially in healthy individuals or those with lung diseases.^3^ In patients with pulmonary diseases, a positive correlation (r = 0.76) was observed between the 6MST and the 6-minute walk test^4^ (6MWT), a test that requires more space to be carried out.^5^ Interestingly, correlation data between the 6MST and the direct measurement of functional capacity are scarce, especially in patients with cardiovascular diseases.

The study by Ritt et al.^6^ seeks to fill this gap by submitting 171 patients with heart failure and coronary artery disease to the 6MST and to the cardiopulmonary test on a treadmill. The results showed a significant correlation between VO_2_peak obtained in the treadmill test and the performance at the 6MST (r = 0.69). Moreover, a prediction equation for VO_2peak_ estimation was developed for men [VO_2peak_ = 19.6 + (0.075*6MST) - (0.10*age)] and for women [VO_2peak_ = 19.6 + (0.075*6 6MST) - (0.10*age) - 2], based on the step test results. Finally, the authors identified 105 climbs as the cutoff point for VO_2peak_ > 20 mL/kg*min, which in cardiac patients is considered a good indicator of cardiovascular prognosis.

Despite the interesting results, it is important to highlight aspects that still deserve to be elucidated in future studies. Important psychometric indicators of the 6MST are still lacking, such as reproducibility, sensitivity to change. The identification of cutoff points is highly relevant and applicable in clinical practice. However, to identify individuals with low functional aptitude, it is necessary that new points be established, considering age, gender, height, among other factors. Cutoff points based on a single and heterogeneous sample are possibly identifying those who are older and female. However, it may be that compared to peers of the same age and gender, they have functional capacity within the expected range. These aspects have already been widely discussed regarding the 6-minute walk test and could be considered for the 6MST.^7^^–^^9^

In summary, the work by Ritt et al.^6^ shows interesting initial evidence of the use of the 6MST to assess the functional aptitude in patients with coronary artery disease and heart failure. As this is a test with great potential for use in clinical practice, future studies on the 6MST as a prognostic marker, on its psychometric characteristics, as well as reference values according to gender and age, will be welcome.
